# Themes and Topics on Diversity, Equity, and Inclusion in JMIR Dermatology Publications

**DOI:** 10.2196/48762

**Published:** 2024-02-02

**Authors:** Ramiro Rodriguez, Karima M Osman, Lachlan Anderson, Micah Pascual, Robert P Dellavalle

**Affiliations:** 1 Department of Dermatology University of Colorado Anschutz Medical Campus Aurora, CO United States; 2 Texas Christian University School of Medicine Fort Worth, TX United States; 3 Dermatology Service US Department of Veterans Affairs Rocky Mountain Regional Medical Center Aurora, CO United States; 4 Department of Epidemiology Colorado School of Public Health University of Colorado Anschutz Medical Campus Aurora, CO United States

**Keywords:** diversity, equity, inclusion, editor, DEI, committee, disparity, underrepresented, dermatology, skin of color, SOC

## Abstract

Publications dealing with topics considered to be pertinent to diversity, equity, and inclusion are increasing. Due to the increasing trend, dermatology journals have started to implement ways to evaluate and understand these publications. Here, we discuss a keyword approach to identify and then categorize these publications. Keywords identified 43 manuscripts. Two reviewers screened the articles’ titles and abstracts, and recommended a full manuscript review for 24 publications. Through the scope of definitions from the National Institutes of Health, an editorial board member performed a full-text review and assigned a primary theme to the publications. Themes included equity (n=20) and diversity/inclusion (n=4). Topics were racial/ethnic differences in care delivery or society (n=17), incomplete understanding of gender and sex (n=3), gender identity (n=2), socioeconomic class and its impact on health (n=1), care for rural underserved communities (n=1), and religion (n=1). The results of this review demonstrate a predominance of equity-related publications, particularly emphasizing racial/ethnic differences in health care delivery, in the publications identified in *JMIR Dermatology*. Future research can focus on creating a review aid to assist editorial board members when providing feedback to manuscripts, refining the keywords, and using thematic analysis methodology to evaluate large sets of publications.

## Introduction

Disparities in racial/ethnic diversity within dermatology prevail. Despite the disparity, dermatology journals published more articles on topics related to diversity, equity, and inclusion (DEI) from 2008 to 2019 compared to other specialties [1]. In the absence of a formal DEI review process, publications risk propagating an incomplete understanding of social determinants of health and their interplay with race/ethnicity, gender identity, sex assignment at birth, and religion [2].

To the authors’ knowledge, evidence-based approaches to reviewing manuscripts dealing with DEI topics and the impact of DEI committees or a DEI editorial board member are limited. *The Journal of Vascular Surgery* noted a similar pattern [3], appointed a DEI editor, and subsequently observed an increase in publications on DEI topics. *JAMA Dermatology* published an article [4] describing a DEI framework in editorial reviews, publication diversity, the need for publishing measures/metrics, and future steps required for implementation. The *Journal of the American Academy of Dermatology* (*JAAD*) has also instituted extra review layers for manuscripts exploring sexuality, gender identity, race/ethnicity, religion, or other potentially emotive topics [5]. *JMIR Dermatology* acknowledges the need for understanding these topics. Thus, the purpose of this paper is to improve the understanding of DEI manuscripts and identify themes and topics within publications.

## Methods

### Overview

Previous research defined DEI publications using target keywords [6]. Our diverse team assigned DEI keywords (Textbox 1) and used JMIR Publication’s editorial management system (Open Journal Systems [OJS]) to find and identify 43 potential DEI manuscripts. Two independent reviewers read the abstracts to determine if a dedicated DEI editor would be recommended and the reason for their assessment. Conflicts prompted a third full-text review. A total of 24 manuscripts received a DEI review recommendation. A *JMIR Dermatology* editorial board member then performed a full-text review and categorized each manuscript’s primary theme and topic. The primary theme was selected within the scope of definitions from the National Institutes of Health (NIH) [7] (Textbox 1).

**Key terms and words used to identify and define publications dealing with diversity, equity, and inclusion.**

**Keywords**

Disparities, diversity, equity, inclusion, disparity, underserved, rural, Black, Hispanic, Latinx, Latino, LGBTQ, skin of color, Asian, Pacific Islander, Native American, American Indian, Alaska Native, White, gender, sex, underrepresented in medicine, minority, URM

**Diversity**

The practice of including many communities, identities, races, ethnicities, backgrounds, abilities, cultures, and beliefs of people, including underserved communities.

**Equity**

The consistent and systematic fair, just, and impartial treatment of all individuals, including individuals who belong to underserved communities that have been denied such treatment.

**Inclusion**

The recognition, appreciation, and use of the talents and skills of individuals of all backgrounds.

### Ethical Considerations

Data was publicly available and deidentified, and did not require institutional review board review.

## Results

In the 24 reviewed manuscripts, primary publication themes dealt with equity (n=20), followed by diversity and inclusion (n=4). The topics included racial/ethnic differences in care or society (n=17), incomplete understanding of gender and sex (n=3), gender identity (n=2), socioeconomic class and its impact on health (n=1), care for rural underserved communities (n=1), and religion (n=1).

## Conclusion

DEI publications are more common relative to previous decades. Dermatology journals are incorporating measures to provide evidence-based methods to improve our understanding of DEI publications. Here, we described a way to evaluate DEI publications within *JMIR Dermatology* and their common themes/topics. Limitations of our study include the sample size. The themes of DEI can also overlap among publications. Standard definitions of DEI assisted the primary theme assignment. Based on the definitions adapted from the NIH, diversity is characterized by including individuals. Inclusion is distinguished by recognizing and appreciating them. Equity was the most prevalent theme and highlights the fair, just, and equal treatment of individuals in the scope of bias. While our authors are diverse, our perspectives are limited and may not be inclusive of all themes or topics within DEI literature. Future research can focus on creating a DEI review aid for editorial boards, broadening and refining the keywords, and using thematic analysis methodology to identify themes/topics among larger sets of publications.

## Abbreviations

DEI: diversity, equity, and inclusion
JAAD: *Journal of the American Academy of Dermatology*
NIH: National Institutes of Health
OJS: Open Journal Systems
